# Our Original Department

**Published:** 1872-11

**Authors:** 


### PART III.

### Editorials, Correspondence and Miscellaneous.

### OUR ORIGINAL DEPARTMENT.

Our readers will be pleased, we think, with the Original Articles in the present number. We hope these friends will continue to favor us and our readers. We will always be glad to hear from them. We take this occasion to invite our readers and friends everywhere to send us communications. Friends, will you not remember this request, and write often? We hope so.

